# Safety and Feasibility of the Plantar Portal Technique in the Surgical Management of Plantar Fasciitis: A Cadaveric Study

**DOI:** 10.1002/jfa2.70105

**Published:** 2025-12-20

**Authors:** Edip Yilmaz, Tahir Koray Yozgatli, Alp Aktekin, Ozer Ozturk, Mustafa Aktekin, Baris Kocaoglu

**Affiliations:** ^1^ Department of Orthopedics and Traumatology Acibadem Altunizade Hospital Istanbul Turkey; ^2^ Department of Orthopedics and Traumatology, School of Medicine Acibadem Mehmet Ali Aydinlar University Istanbul Turkey; ^3^ Bahcesehir University School of Medicine Istanbul Turkey; ^4^ Department of Anatomy, School of Medicine Acibadem Mehmet Ali Aydinlar University Istanbul Turkey

**Keywords:** endoscopic surgery, plantar fascia release, portal safety

## Abstract

**Introduction:**

This study investigates the proximity of the portals and nearby motor and sensory nerves in endoscopic release of the plantar fascia and the safety and efficacy of the addition of a novel third (plantar) portal to improve access to the calcaneal spur.

**Methods:**

Nine fresh‐frozen lower extremity cadavers were examined in this study. In addition to the conventional medial and lateral portals for endoscopic plantar fascia release, a third (plantar) portal was utilized to debride the calcaneal spur area. The fascia was released with the use of an obturator cannula. Each cadaver was carefully dissected, the distance of the nerves to the portals and the obturator cannula was measured with a digital caliper, and the adequacy of the plantar fascial cut was evaluated. The nerves were examined along their course to check for any injuries.

**Result:**

The closest distance between the lateral plantar nerve and the plantar portal was 15.3 ± 1.47 mm, and the obturator cannula was 10.53 ± 1.50 mm. The closest distance between the first branch of the lateral plantar nerve (Baxter's nerve) and the obturator cannula was 10.02 ± 0.65 mm. The closest distance between Baxter's nerve and the calcaneal spur area was 8.21 ± 1.12 mm. There was no evidence of nerve or muscle injury in the dissected cadavers.

**Conclusions:**

Using the plantar portal in conjunction with conventional portals in endoscopic plantar fasciitis surgery can be advantageous, as it provides easier access to the calcaneal spur and is safe in terms of its distance from the nearby neural structures.

## 1 Introduction

Plantar fasciitis is characterized by enthesopathy at the area of plantar fascia attachment to the medial tubercle of the calcaneus [1]. Patients often experience pain, particularly during their first steps in the morning or after intense physical activity. Radiographs may reveal a bony growth frequently referred to as a heel spur. It is postulated that the heel spur does not directly cause pain but rather contributes to pain by causing irritation and degeneration in the plantar fascia due to its location [2, 3].

Conservative treatment consisting of nonsteroidal anti‐inflammatory medications, shoe modification, and physical therapy is the first treatment option, and approximately 80% of patients benefit from these interventions [1, 4, 5]. However, the persistence of severe pain despite conservative therapy and the resulting limitations on the individual's physical activity indicate the need for surgical intervention. Surgical techniques, such as complete or partial plantar fascia release, can be performed through either open or endoscopic surgery. Plantar fascia release is now more commonly performed endoscopically [6]. However, endoscopic surgery presents some unique risks and challenges due to the use of special instruments and limited anatomical exposure. Specifically, the proximity of portals to sensory nerves and muscles, as well as the risk of damaging these structures during plantar fascia release, poses potential threats. The demanding technique for complete heel spur excision, utilizing conventional medial and lateral portals, and the long learning curve associated with the endoscopic surgical technique pose important challenges. It is unknown whether a third (plantar) portal could safely assist in resecting the calcaneal spur.

This study aimed to evaluate the proximity of the traditional medial and lateral portals and the nearby sensory nerves, and the potential benefit of adding a plantar portal to the plantar surface for easier access to the heel spur. The hypothesis was that using the plantar portal in endoscopic plantar fasciitis surgery and spur resection would be advantageous, providing easier access to the calcaneal spur and offering a safe option in terms of its distance from nerves and surrounding muscles.

## 2 Materials and Methods

Nine fresh‐frozen lower extremity cadavers were utilized for this study. All cadaveric specimens were used in compliance with ethical regulations. Donor parts were frozen within 24–48 h after death and stored at −20°C until use. Thawing was performed at 4°C for 12 h, followed by room temperature for 12 h to restore tissue pliability. All procedures were conducted in a cadaveric operating room at 20°C using standard surgical instruments and manufacturer‐recommended energy settings. No chemical preservatives were used to preserve tissue handling characteristics.

The inclusion criteria were fresh‐frozen cadaveric feet with normal bony anatomy, no history of prior surgery involving the feet, no history of previous trauma affecting foot anatomy, and no signs of talocalcaneal joint osteoarthritis. The exclusion criteria were prior surgery or trauma that caused scarring to the feet, which could affect the distance measurements of the neural structures to the portals; abnormal gross anatomy leading to deformed bony architecture, which could result in aberrant portal placement; and any other condition that could compromise the accuracy of the measurements. The length of each cadaveric foot was measured and recorded.

### 2.1 Ethics Statement

All cadaveric study procedures were performed in strict accordance with the ethical principles established by the institutional review board. In our institution, body donors provide informed consent during their lifetime for the use of their bodies in education and research. Therefore, separate approval from the ethics committee is not required for each individual cadaveric study. The authors sincerely thank those who donated their bodies to science, allowing anatomical research to be performed. Results from such research can potentially increase mankind's overall knowledge, which can then improve patient care. Therefore, these donors and their families deserve our highest gratitude [7].

### 2.2 Medial and Lateral Portal Creation

**FIGURE 1:**
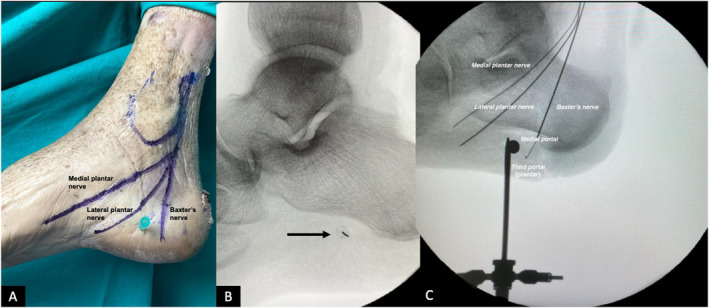
Cadaveric image and fluoroscopic images showing the medial and plantar portal placements along with trajectories of the nerves. (A) Cadaveric image of the medial side of a right foot specimen showing the approximate trajectories of the nerves (purple lines) and point of medial portal placement (green syringe needle). (B) Fluoroscopic image confirming correct placement of the portal site. An arrow marks the target site of the portal. (C) Obturator cannula in the medial portal and trocar in the plantar portal (positioned perpendicular to the obturator). The nerves are traced with thin metal wires to show their proximity to the portals.

**FIGURE 2:**
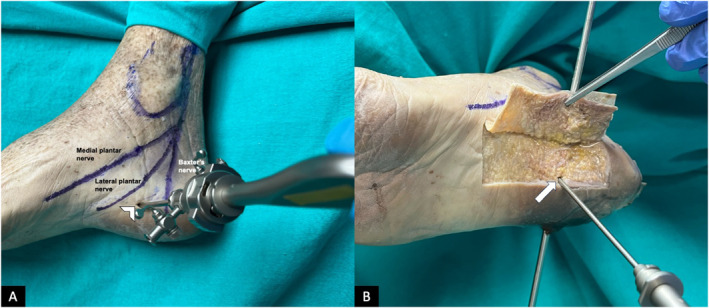
Placement of the medial portal and plantar portal in a right foot cadaveric specimen. (A) Medial side of the foot, showing the medial portal (arrowhead) with the trocar in place. (B) Mid dissection showing the reflected skin, subcutaneous fat overlying the plantar fascia, the medial‐lateral portal line, and the plantar portal (arrow) placed at a right angle to it. The obturator is placed in from the medial portal through the foot and out from the lateral portal.

Three portals were created in each cadaveric foot: one medial, one lateral, and one plantar portal at a non‐weight‐bearing area. Firstly, to identify the optimal placement of the medial portal, a point 10‐mm distal to the calcaneal tubercle and 5 mm above the plantar surface of the foot was determined under fluoroscopy (Figures 1A,B and 2A). Then, a skin incision was made with a size 11 scalpel; care was taken not to damage any underlying deep structure. The medial portal was dissected bluntly with a surgical clamp. Then, the endoscopic obturator (EndoBlade Soft Tissue Release System, Arthrex Inc., Naples, FL, USA) was advanced bluntly from medial to lateral between the plantar fascia and plantar fat tissue. It is important to advance the trocar bluntly, and avoid sharp dissection blindly, not to damage the lateral plantar nerve and its first branch, Baxter's nerve. The obturator was advanced up to the lateral subcutaneous tissue, and the position of the lateral portal was determined from the skin bulge. A lateral portal was opened with a size 11 scalpel, and the obturator was advanced from the lateral portal.

### 2.3 Endoscopic Plantar Fascia Release

A crescent‐shaped clear cannula was placed over the obturator from the lateral to the medial portal. The endoscope device was advanced through the cannula through the lateral portal, and the plantar fascia was visualized. The triangular knife was introduced through the medial portal and advanced to the lateral border of the plantar fascia. Then, the plantar fascia was released endoscopically from lateral to medial with a triangular knife.

### 2.4 Plantar Portal Creation and Calcaneal Spur Excision

After the release of the plantar fascia, a skin incision was made with a number 11 scalpel for the plantar portal. The location of the incision is at a point 10‐mm distal to the calcaneal tubercle at the level of the skin overlying the trocar in the middle part of the plantar surface of the foot (Figures 1C and 2A,B). Then, while visualizing from the medial portal and using a shaver and a radio frequency device from the plantar portal and from the lateral portal as needed, a working area was created between the plantar fascia and the flexor digitorum brevis, and the calcaneal spur was exposed. In cases where needed, a small retractor was advanced from the lateral portal to help with exposure, whereas the working instruments were in the plantar portal. During this procedure, care was taken to make sure the instruments were working facing the bone while performing the endoscopic dissection, and the radio frequency device was used intermittently not to damage the Baxter's nerve, which is located just above the flexor digitorum brevis. The instruments are introduced at a slightly oblique angle to reach the calcaneal spur, and staying below the muscle is critical as the nerve is protected by the muscle layer above. This would ensure a muscular layer of FDB, about 10 mm thick and adequate to protect the nerve [8]. After debridement of the proximal portion of the plantar fascia and any soft tissue covering the calcaneal spur, it was confirmed under fluoroscopy that the instruments were in contact with the calcaneal spur, and the calcaneal spur was removed with a burr device. The removal of the bony prominence in the spur area was confirmed endoscopically and fluoroscopically. The same standardized surgical technique was repeated for each cadaveric foot.

### 2.5 Anatomical Dissection and Measurements

**FIGURE 3:**
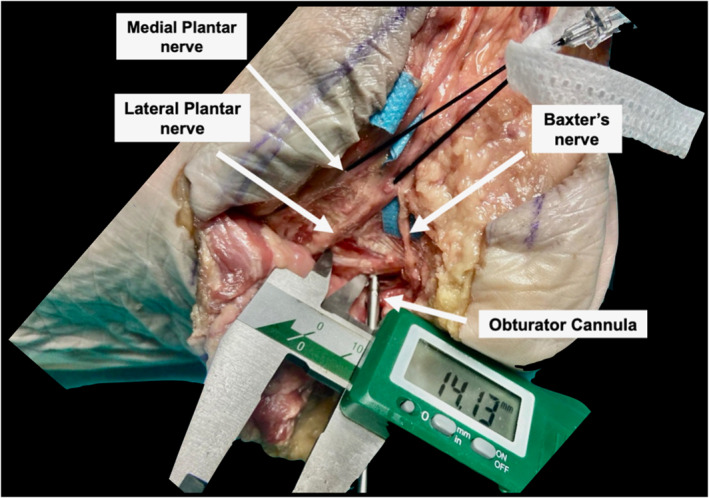
Cadaveric dissection and measurement of the distance from the obturator cannula to the lateral plantar nerve. Arrows show the medial plantar nerve, the lateral plantar nerve, and Baxter's nerve.

After the completion of the procedures, each cadaveric foot was carefully dissected to analyze the spatial relationships between the portals and the cannula, and the lateral plantar nerve and its first branch, the Baxter's nerve. The dissections were performed by a foot and ankle specialist orthopedic surgeon and an anatomy professor, each with 20 or more years of experience in their fields. The successful release of the plantar fascia was confirmed with the dissection. The posterior tibial nerve was identified posterior to the medial malleolus, and by following the course of the nerve, the medial plantar nerve, the lateral plantar nerve, and the calcaneal branches of the posterior tibial nerve were identified (Figure 3). The closest distances from the portals and the cannula after the obturator's trajectory to the medial and lateral plantar nerves and the first branch of the lateral plantar nerve, the so‐called Baxter's nerve, were measured. All measurements were performed with a digital caliper. For each cadaveric sample, two measurements were made separately by an experienced anatomist and an experienced foot and ankle orthopedic surgeon. The Bland–Altman method was used to calculate the 95% limits of agreement for all measurements, and the mean difference was found to be within 0.5 mm [9].

### 2.6 Statistical Analysis

All measurements were presented as mean ± SD for continuous variables and number (%) for categorical variables. The Bland–Altman method was used to calculate the 95% limits of agreement for all measurements on the cadaveric feet. A power analysis was conducted to determine the required number of cadaveric feet to assess third portal's safety during endoscopic plantar fascia release by examining the distance to sensory nerves. With a defined safety threshold of 8 mm and assuming an expected mean distance of 10 mm with a standard deviation of 1 mm, the calculated effect size (*d* = 2.0) indicated a substantial difference from the threshold. A one‐tailed *t*‐test (*α* = 0.05) with desired power levels of 0.80 and 0.90 suggested sample sizes of approximately 6–10 and 8–12 cadaveric feet, respectively. Based on this analysis, we selected a sample size of 9 cadaveric feet for this study. This was deemed appropriate to provide sufficient power to confidently evaluate the plantar portal's safety. All statistical analyses were performed using *R* statistical software (R version 4.2.1; *R* Foundation for Statistical Computing).

## 3 Results

The mean closest distance between the lateral plantar nerve and the plantar portal was 15.21 ± 1.42 mm. The mean closest distance between Baxter's nerve and the plantar portal was 10.46 ± 0.66 mm. The mean closest distance between the lateral plantar nerve and the obturator cannula was 10.61 ± 1.56 mm. The closest distance between Baxter's nerve and the obturator cannula was 10.32 ± 0.82 mm. The closest distance between Baxter's nerve and the tuber calcanei was 8.37 ± 1.06 mm (Table 1).

**TABLE 1:** Closest distances measured from the plantar portal and cannula (i.e., placed between the medial and lateral portals and the tuber calcanei to the sensory nerves).

**Cadaver number**	**Plantar portal to Baxter's N. (mm)**	**Plantar portal to lateral plantar N. (mm)**	**Cannula to lateral plantar N. (mm)**	**Cannula to Baxter's N. (mm)**	**Tuber calcanei to Baxter's N. (mm)**
1	10.71	14.93	9.07	10.24	7.36
2	11.30	17.48	9.89	10.86	9.20
3	10.17	13.20	10.70	9.87	6.39
4	10.85	16.74	13.59	10.40	9.70
5	9.15	15.87	10.61	8.79	8.56
6	10.70	14.16	10.54	9.78	8.36
7	9.88	13.54	9.20	11.51	9.50
8	11.04	15.70	12.58	11.24	8.42
9	10.36	15.26	9.30	10.23	7.87
**Mean**	**10.46**	**15.21**	**10.61**	**10.32**	**8.37**
**SD**	**0.66**	**1.42**	**1.56**	**0.82**	**1.06**

Abbreviation: SD, standard deviation.

**FIGURE 4:**
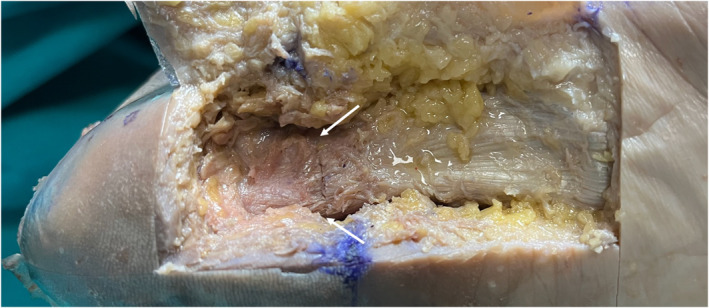
Dissection of a cadaveric specimen showing the endoscopic cut made on the plantar fascia (arrows). This specimen had a 100% cut along the whole width of the fascia.

The average length of the feet in the study was 22.8 ± 0.9 cm. The average cut percentage of the plantar fascia was 85.1 ± 8.1% (Table 2, Figure 4).

**TABLE 2:** Foot length and fascia width measurements.

**Cadaver** **number**	**Side**	**Foot length (cm)**	**Width of the plantar fascia (mm)**	**Cut length (mm)**	**Cut** **percentage (%)**
1	L	22.20	26.20	22.50	85.9
2	R	23.40	24.70	20.00	81.0
3	R	21.20	19.60	14.30	73.0
4	L	22.80	21.00	21.00	100.0
5	L	23.90	16.10	13.70	85.1
6	L	22.30	17.50	14.50	82.9
7	L	23.70	18.60	15.00	80.7
8	R	23.50	14.80	12.20	82.4
9	L	22.10	15.20	14.50	95.4
**Mean**		**22.79**	**19.30**	**16.41**	**85.1**
**SD**		**0.90**	**4.04**	**3.71**	**8.1**

Abbreviations: L, left; R, right; SD, standard deviation.

## 4 Discussion

The most significant finding of the study was that the use of the plantar portal for endoscopic plantar fasciitis surgery with calcaneal spur resection is advantageous, as it provides easier access to the heel spur and offers a safer option due to its distance from the nerves. The closest distance between the lateral plantar nerve and the plantar portal was 10.46 ± 0.66 mm, and the closest distance between Baxter's nerve and the plantar portal was 15.21 ± 1.42 mm, making this portal a safe option.

Approximately 10% of patients may not experience pain relief despite at least 1 year of conservative treatment of plantar fasciitis, and these patients may eventually require surgical intervention [10, 11]. Complications of open surgery, such as infection, skin problems, and prolonged recovery, have led to the development and widespread use of endoscopic surgery [6, 12]. Barrett and Day first introduced the plantar fascia release with a single‐portal endoscopic surgical technique [13]. O'Malley et al. reported that the mean AOFAS score improved from 62 to 80 points in 20 patients treated with partial endoscopic plantar fascia release using a superficial medial and lateral portal [14]. However, accessing the calcaneal spur is difficult with this technique. Fumito Komatsu et al. described the deep medial portal technique [15]; although this technique provides easier access to the calcaneal spur, the proximity of this deep medial portal to the neural structures could be an important disadvantage.

The effectiveness of calcaneal spur excision in plantar fasciitis surgery is still controversial [3], and the presence of a calcaneal spur alone may not directly lead to heel pain [16]. It was shown that there is high blood flow to this area and many nerve terminals near the calcaneal spur. In addition, the presence of a calcaneal spur can cause further degeneration due to irritation of the plantar fascia [17]. In this context, it is plausible that excision of the calcaneal spur would be beneficial. Because of technical difficulties associated with previously described techniques, a novel approach is needed to facilitate this step of the surgery. In this study, it was hypothesized that plantar fascia release could be performed with the superficial medial and lateral portal technique, and then calcaneal spur excision could be performed with a third portal using arthroscopic instruments.

In endoscopic plantar fascia release, the lateral plantar nerve and its first branch, the Baxter's nerve, and surrounding muscle structures are at risk due to their proximity to the portals and, more importantly, to the calcaneal spur. In this study, the proximity of the medial portal to the nerves was found to be similar to the results reported in the literature [6, 18]. Additionally, it was observed that the plantar portal was slightly farther from the nerves than the medial portal. However, considering the possibility of anatomical variations, care should be taken when performing endoscopic plantar fascia release. Although it was thought that it would be easier to reach the calcaneal spur from the plantar portal, the location of the incision on the plantar side could be a disadvantage due to the possibility of wound problems. However, by placing the portal incision at a non‐weight‐bearing area of the plantar surface, this risk could be minimized. Furthermore, we found the proximity of the Baxter's nerve to the calcaneal medial tubercle to be 8.22 mm, which indicates that the risk of injury to the nerve during calcaneal spur excision is high. We think that resection should not be performed under the flexor digitorum brevis and laterally, primarily because the Baxter's nerve is located just superior to the flexor digitorum brevis muscle and surfaces laterally to enter the abductor digiti minimi muscle. Although this may lead to partial resection of the calcaneal spur, it is preferable to avoid potential neural damage; in this study, the nerve was not damaged near the spur.

Because complete release of the plantar fascia may lead to disturbance of the longitudinal arch and dorsolateral foot pain due to excessive loading of the lateral column, Brugh et al. suggested that the fascia should not be loosened more than 50% [19]. However, the clinical effect of the amount of loosening is still unknown, and some authors aim for higher release percentages. In our study, we initially aimed to loosen the plantar fascia by 50%. However, when the cadavers were dissected, it was seen that none of them were around 50%, and the average loosening was measured as 85%. This situation shows that the loosening performed endoscopically was more than expected. Endoscopic release techniques are highly effective, and less aggressive loosening of the fascia could be utilized in clinical practice to achieve clinically effective results. Direct visualization and preservation of the lateral plantar fascia (abductor fascia) may help prevent the consequences of excessive resection of the plantar fascia.

This study is not without its limitations. Firstly, this was an anatomical cadaveric study that aimed to evaluate the safety and effectiveness of the plantar portal technique to perform heel spur resection. However, the foot is a dynamic structure, and the healing response and clinical outcomes are crucial in determining the effectiveness of new methods. Thus, future clinical studies with a medium‐ to long‐term follow‐up are needed to evaluate the in vivo safety and effectiveness of this technique. Secondly, we observed that the plantar fascia was released more than initially planned with the current surgical technique. In the clinical setting, it is not possible to evaluate the amount of fascia release; however, less aggressive release of the fascia with sparing of up to 50% of the length under direct visualization should be aimed for in surgery. Thirdly, surgeons must be aware of the potential for variations in the normal anatomy and that, despite all precautions, nerve damage may occur with the described technique, as in any other surgery. All patients must be informed regarding the risk of damage to neural structures before any surgery. Lastly, this was an anatomical study with no biomechanical measurements. The biomechanical outcomes of the endoscopic plantar fascia release were beyond the scope of this study; however, future research could be used to evaluate the changes in the loading of the foot with endoscopic plantar fascia release with heel spur removal using the plantar portal.

## 5 Conclusion

The use of the plantar portal in endoscopic plantar fasciitis surgery and spur resection is considered advantageous because it provides easier access to the heel spur and offers a safe option in terms of its distance from the nerves. The distances from the third plantar portal to the lateral plantar and Baxter nerves are at a mean of 15.21 and 10.46 mm, respectively, which makes this portal a safe option. This study presents a novel approach to reevaluate the proximity of the traditional medial and lateral portals and the nearby sensory nerves and adds a third plantar portal to the plantar surface for easier access to the heel spur.

## Author Contributions

**Edip Yilmaz:** conceptualization, data curation, formal analysis, investigation, writing – original draft, writing – review and editing. **Tahir Koray Yozgatli:** investigation, data curation, methodology, formal analysis, visualization, writing – original draft, writing – review and editing. **Alp Aktekin:** investigation, data curation, visualization, writing – review and editing. **Ozer Ozturk:** investigation, writing – review and editing. **Mustafa Aktekin:** conceptualization, investigation, writing – original draft, writing – review and editing. **Baris Kocaoglu:** conceptualization, investigation, writing – original draft, writing – review and editing, supervision.

## Funding

The authors have nothing to report.

## Ethics Statement

Ethical approval was waived for this cadaveric study.

## Conflicts of Interest

The authors declare no conflicts of interest.

## Permission to Reproduce Material From Other Sources

The authors have nothing to report.

## Supporting information


Supporting Information S1

## Data Availability

Data from this study are available from the corresponding author upon reasonable request.
