# Supplementary material for: Safety and Feasibility of the Plantar Portal Technique in the Surgical Management of Plantar Fasciitis: A Cadaveric Study
Source: J Foot Ankle Res. 2025 Dec 20;18(4):e70105. doi: 10.1002/jfa2.70105 (PMC12718177; doi:10.1002/jfa2.70105)
Supplement: Supplementary file 1 — Supporting Information S1 [file JFA2-18-e70105-s001.docx]

**CACTUS guidelines.**

**1 Approval of the use of the bodies with a clear statement of the institute that approves their use.**

- All procedures for this cadaveric study were performed in strict accordance with the ethical principles established by the institutional review board. In our institution, body donors provide informed consent during their lifetime for the use of their bodies for educational and research purposes. No separate approval from the local ethics committee is required for each individual cadaveric study.
- The authors sincerely thank those who donated their bodies to science so that anatomical research could be performed. Results from such research can potentially increase mankind’s overall knowledge that can then improve patient care. Therefore, these donors and their families deserve our highest gratitude.

**2 Number and gender of the bodies and/or organs used. If possible, report important clinical data such as BMI, basic medical history or previous non neglectable surgery.**

- Nine cadaveric feet were used in the study. There were no previous surgery performed on the feet. The BMI data and medical history other than the lack of surgery and trauma were not available.

**3 State of conservation of corpses and/or parts of them, indicating the days of death before preservation.**

- Donated corpses are accepted to the department of anatomy immediately after death.
- All corpses in this study are fresh frozen, time elapsed from the conservation procedure to the use of corpse for study purposes is between 3-6 months.

**4 In case of preparation and/or embalming of the body/organ, briefly indicate the methodology used (i.e. Fresh frozen, Thiel’s technique, etc) and the time elapsed from the conservation procedure to the use of the corpse for training/study purposes.**

- Donated corpses are accepted to the department of anatomy immediately after death.
- All corpses in this study are fresh frozen, time elapsed from the conservation procedure to the use of corpse for study purposes is between 3-6 months.

**5 Indicate the type of study for which they are used (anatomical study, surgical study, surgical training, device training, etc).**

- The cadaveric samples were used for a surgical study.

**6 The type of fluids other than water with which the bodies come into contact during the study (i.e.Saline solution 0.9%, formaldehyde, etc) might be a useful additional but not mandatory information.**

- These fresh frozen samples did not come into contact with any fluids during the study period, only saline was used during endoscopic surgery.

**7 If cadaver specimens are sampled for pathological evaluation, the type and method of sampling might be a useful additional but not mandatory information.**

- Pathological evaluation was not performed, gross cadaveric dissection was performed on each sample.

**8 Indicate the number and qualification of investigators/trainees actively involved in the cadaver study/training. Only people involved in organization and training should be present in the activities, since the use of human bodies for study should be treated with all the ethics they deserve.**

- One anatomy professor, one orthopedic surgery professor, one consultant orthopedic surgeon, one orthopedic surgery resident and one medical student were involved in the investigation.

**9 Provide brief outcomes in terms of satisfaction in the use of the cadaver model through a short questionnaire to be administered to the trainees/investigators and comparing the different models used in the study (i.e. Thiel fixed cadavers vs fresh frozen; i.e. human cadaver model used vs another biological or non-biological model used). If the human cadaver is the only model used in the study provide an overall comment on satisfaction compared to that expected. When it is possible, also report objective data on the usefulness of the training model.**

- Based on the feedback from the investigators the use of the cadaveric samples were justified and the utilization of the cadaveric feet was the only method to provide adequate safety evaluation for the surgical technique proposed in the study.
